# SARS-CoV-2 hijacks cellular kinase CDK2 to promote viral RNA synthesis

**DOI:** 10.1038/s41392-022-01239-w

**Published:** 2022-12-27

**Authors:** Saisai Guo, Xiaobo Lei, Yan Chang, Jianyuan Zhao, Jing Wang, Xiaojing Dong, Qian Liu, Zixiong Zhang, Lidan Wang, Dongrong Yi, Ling Ma, Quanjie Li, Yongxin Zhang, Jiwei Ding, Chen Liang, Xiaoyu Li, Fei Guo, Jianwei Wang, Shan Cen

**Affiliations:** 1 Department of Immunology, Institute of Medicinal Biotechnology, Chinese Academy of Medical Sciences & Peking Union Medical College, Beijing, China; 2 NHC Key Laboratory of Systems Biology of Pathogens and Christophe Mérieux Laboratory, Institute of Pathogen Biology, Chinese Academy of Medical Sciences & Peking Union Medical College, Beijing, China; 3 Beijing Key Laboratory for Pediatric Diseases of Otolaryngology, Head and Neck Surgery, Key Laboratory of Major Diseases in Children, Ministry of Education, Beijing Pediatric Research Institute, Beijing Children’s Hospital, Capital Medical University, National Center for Children’s Health, Beijing, China; 4 Lady Davis Institute, Jewish General Hospital, McGill University, Montreal, QC Canada; 5 CAMS Key Laboratory of Antiviral Drug Research, Chinese Academy of Medical Sciences & Peking Union Medical Sciences, Beijing, China

**Keywords:** Infection, Microbiology, Drug screening, Infectious diseases

## Abstract

The coronavirus disease 2019 (COVID-19) pandemic has devastated global health. Identifying key host factors essential for SARS-CoV-2 RNA replication is expected to unravel cellular targets for the development of broad-spectrum antiviral drugs which have been quested for the preparedness of future viral outbreaks. Here, we have identified host proteins that associate with nonstructural protein 12 (nsp12), the RNA-dependent RNA polymerase (RdRp) of SARS-CoV-2 using a mass spectrometry (MS)-based proteomic approach. Among the candidate factors, CDK2 (Cyclin-dependent kinase 2), a member of cyclin-dependent kinases, interacts with nsp12 and causes its phosphorylation at T20, thus facilitating the assembly of the RdRp complex consisting of nsp12, nsp7 and nsp8 and promoting efficient synthesis of viral RNA. The crucial role of CDK2 in viral RdRp function is further supported by our observation that CDK2 inhibitors potently impair viral RNA synthesis and SARS-CoV-2 infection. Taken together, we have discovered CDK2 as a key host factor of SARS-CoV-2 RdRp complex, thus serving a promising target for the development of SARS-CoV-2 RdRp inhibitors.

## Introduction

The COVID-19 pandemic is caused by the coronavirus SARS-CoV-2, and has gravely burdened global health. SARS-CoV-2 belongs to Coronaviridae and is an enveloped, single-stranded positive-sense RNA virus.^1–3^ Like other coronaviruses, SARS-CoV-2 genome encodes 16 nonstructural proteins (nsps) in orf1a and 1b, 4 structural proteins, as well as several accessory proteins.^4,5^ SARS-CoV-2 has undergone continuous evolution, giving rise to highly transmissible variants of concern (VOCs), such as Delta and Omicron strains, that are less protected by the licensed COVID-19 vaccines and resistant to monoclonal antibody-based therapies.^6–8^ Thus, there has been an urgent, constant need for effective anti-SARS-CoV-2 drugs to mitigate the ongoing COVID-19 pandemic, and also prepare for future viral outbreaks.

Replication of viral RNA is essential for SARS-CoV-2 propagation, which is driven by viral RNA-dependent RNA polymerase (RdRp). The RdRp complex consists of three components: nsp7, nsp8, and nsp12.^9^ Nsp12 is a 932 amino acids long, primer-dependent RNA polymerase, contains the nidovirus RdRp-associated nucleotidyl transferase (NiRAN) domain at N-terminus and a right-hand RdRp domain at C-terminus, which were connected by an interface domain.^10^ Nsp8 functions as a RNA primase, generates short oligonucleotide primers for nsp12.^11,12^ Its essential function in SARS-CoV-2 replication has made the RdRp complex the mostly sought drug target and has been intensively investigated, especially its structure and interaction with cellular factors.

Multiple host proteins have been identified to associate with viral RdRp by means of affinity-purification mass spectrometry (AP-MS).^13,14^ Recent studies revealed that apart from its polymerase activity, the SARS-CoV-2 nsp12 can hijack host RIPK1 (Receptor-interacting serine/threonine-protein kinase 1)^15^ and suppress the nuclear translocation of IRF3 (Interferon regulatory factor 3)^16^ to antagonize host antiviral innate immunity so as to promote viral infection. Moreover, viral RdRp was shown to interact with METTL3 (Methyltransferase 3), regulate its sumoylation and ubiquitination, thus affecting its localization and expression, and regulating the m6A modification of SARS-CoV-2 RNA.^17^ However, very little is known about whether and how SARS-CoV-2 RdRp activity is regulated by host proteins.

In this study, we have sought host proteins that associate with the RdRp of SARS-CoV-2 by means of mass spectrometry (MS) based proteomic approach, and found that CDK2, a member of cyclin-dependent kinases, interacted with nsp12 and induced nsp12 phosphorylation at amino acid T20, thus facilitating the assembly of the RdRp complex and promoting viral RNA synthesis. As a result, CDK2 inhibitors potently inhibited viral RNA synthesis and SARS-CoV-2 replication. CDK2 is a protein kinase that plays key role in regulating cell division.^18^ It has been shown that CDK2 can modify viral proteins and create an environment conducive to virus replication, either by counteracting cellular restrictionor by disrupting cell cycle progression.^19–22^ Our data thus reveals a new function of CDK2 in regulating the function of viral RdRp, and suggests CDK2 as a target for the development of viral RdRp inhibitors.

## Results

### CDKs support SARS-CoV-2 RdRp-mediated gene expression

**Fig. 1:**
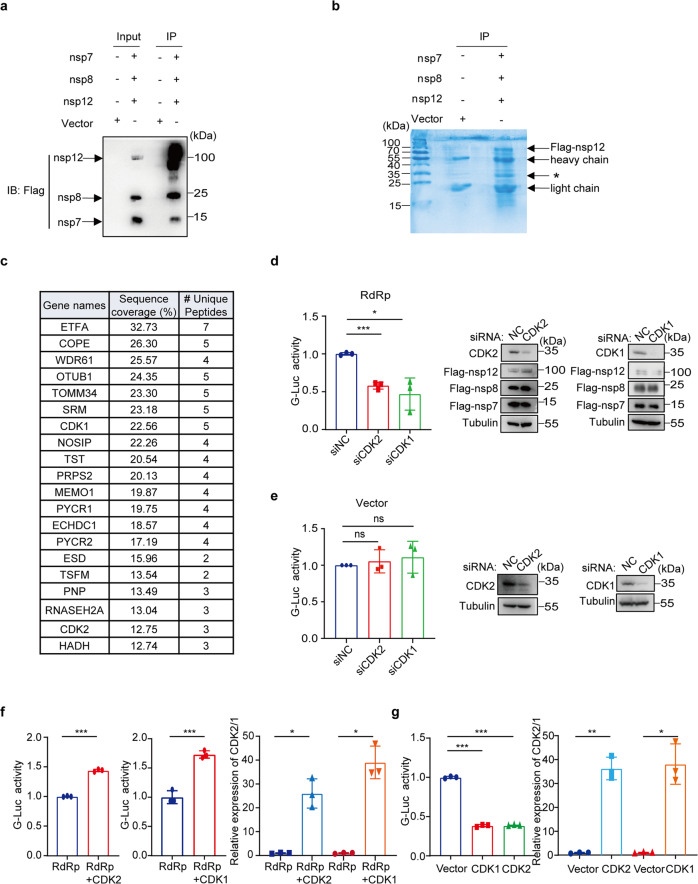
CDKs involve in the activity of SARS-CoV-2 RdRp. **a** Flag-tagged nsp7, nsp8, nsp12, or Flag-vector were co-transfected in HEK293T cells. Anti-Flag M2 affinity gel was used for Co-Immunoprecipitation and Western blot analysis was performed with the indicated antibodies. **b** The immunoprecipitated samples were separated by SDS-PAGE followed by coomassie blue staining. Asterisk (*) indicates the band for mass spectrometry analysis. **c** The top 20 candidate genes indicated by mass spectrometry. **d**, **e** HEK293T cells expressing CoV-Gluc, nsp12, nsp7, nsp8 plasmid DNA at the ratio of 1:10:30:30 (**d**) or control vector and CoV-Gluc (**e**) were transfected with CDK1 or CDK2 siRNA (three siRNAs per gene) for 48 h. Then the Gluc activity was measured in the supernatants. The CDK1/2 knockdown were determined by western blot analysis. **f**, **g** HEK293T cells expressing CoV-Gluc, nsp12, nsp7, nsp8 plasmid DNA at the ratio described above (**f**) or control vector and CoV-Gluc (**g**) were transfected with CDK2/CyclinA plasmids or CDK1/CyclinB plasmids for 48 h. Gluc activity was measured in the supernatants. CDK1/2 overexpression was detected by qRT-PCR. The experiments was performed at least three times in **d**–**g**, and data are presented as mean ± SD; *P < 0.05, **P < 0.01, ***P < 0.001 and ns not significant (two-tailed unpaired Student’s *t*-test)

To identify cellular proteins that modulate the SARS-CoV-2 RNA synthesis, we first performed immunoprecipitation coupled with mass spectrometry (MS) to determine cellular proteins that associate with SARS-CoV-2 RdRp. Briefly, Flag-tagged nsp7, Flag-tagged nsp8, and Flag-tagged nsp12 plasmids were co-transfected into HEK293T cells, and the Flag-tagged proteins in cell lysates were pulled down using anti-Flag-M2 beads. The expression of nsp7, nsp8, and nsp12 in co-transfected cells was detected by western blot (Fig. 1a). The immunoprecipitate samples were loaded for SDS-PAGE, followed by Coomassie blue staining. In addition to viral nsp7, nsp8, and nsp12, visible protein bands appeared at ~35 KD position in the immunoprecipitated samples (Fig. 1b), which were extracted for liquid chromatography-mass spectrometry (LC-MS) analysis. Eighty-four host proteins of molecular weights between 30 KD and 40 KD were identified (supplementary Table S1).

Notably, two cyclin-dependent kinases (CDKs), CDK1 (Cyclin-dependent kinase 1) and CDK2, were among the top 20 hits (Fig. 1c). CDKs have been showed to modulate replication of RNA viruses through regulating cell cycle progression, overcoming cellular restriction factors, and associating with viral RNA polymerase.^19,20,23^ We thus speculated that SARS-CoV-2 may hijack cellular CDKs to promote viral RNA replication.

In agreement with our hypothesis, a recent multi-omics study revealed that many kinases of the CDK family mediated signal transduction during SARS infection by interacting with each other and served as functional hubs.^24^ More importantly, several specific CDK inhibitors are effective in treating cancer.^25^ These CDK inhibitors could be repurposed as effective therapeutics to treat SARS-CoV-2 infections.^25^ Therefore, we selected CDK for further investigation in this study. To test this, we first measured the effect of CDKs knockdown on SARS-CoV-2 RNA synthesis using a CoV-RdRp-Gluc reporter assay system as described previously.^26^ In this assay, the luciferase gene contains the 5’ and 3’ untranslated regions (UTRs) of SARS-CoV-2 and its expression is driven by a CMV promoter. When Gluc mRNA is expressed, the viral UTRs allows this mRNA to be recognized and amplified by viral RdRp, resulting in a substantial increase of Gluc expression, which reports the activity of SARS-CoV-2 RdRp. Upon knockdown of CDK1 or CDK2 with siRNA as shown by western blot analysis, levels of luciferase activity significantly reduced (Fig. 1d), whereas no significant change in luciferase activity was observed in control cells that did not express viral RdRp (Fig. 1e). These data suggest a positive role of CDK2 and CDK1 in the function of SARS-CoV-2 RdRp. We next overexpressed either CDK2 or CDK1, and observed 1.5 fold increase in luciferase activity in cells expressing viral RdRp (Fig. 1f, Supplementary Fig. 1c), and a significant decrease of luciferase in control cells (Fig. 1g, Supplementary Fig. 1d). In addition, we examined whether other candidate genes such as *ETFA*, *OTUB1*, and *CDK5* (another member of the CDK family) also participated in SARS-CoV-2 RdRp-mediated RNA synthesis. The result showed that silencing either of these genes with siRNA had no significant effect upon the luciferase activity (Supplementary Fig. 1a, b), supporting the specific role of CDK1 and CDK2 in stimulating SARS-CoV-2 RdRp-mediated RNA synthesis.

### CDK2 Interacts with SARS-CoV-2 nsp12

**Fig. 2:**
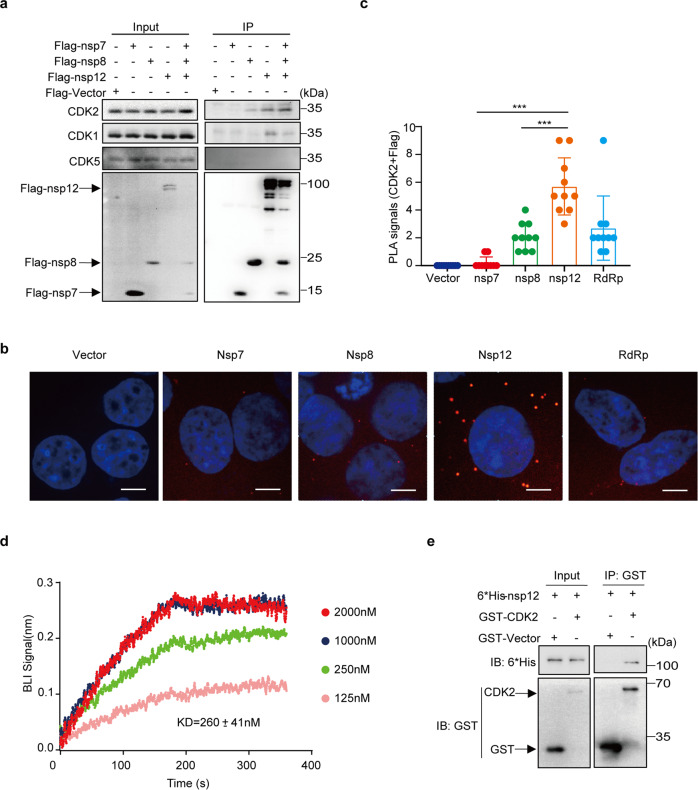
CDK2 interacts with nsp12. **a** Endogenous CDK2 interacts with Flag-nsp7, Flag-nsp8, and Flag-nsp12 in HEK293T cells. Anti-Flag M2 affinity gel was used for Co-Immunoprecipitation and the immunoprecipitates was analyzed by immunoblotting. **b** In situ proximity ligation assay (PLA) and confocal imaging to show the interaction of endogenous CDK2 with Flag-nsp7, Flag-nsp8, Flag-nsp12 (red) in HEK293T cells. Nuclei were counterstained with DAPI (blue). Scale bars, 5 μm. **c** The number of PLA red fluorescent dots were counted in randomly selected cells. *n* = 10 cells per group. Data are presented as mean ± SD. Two-tailed unpaired Student’s *t*-test was applied between group nsp12 and group nsp8. Two-sided Mann–Whitney test was performed between group nsp12 and group nsp7, ***P < 0.001. **d** Octet assay to detect the binding of CDK2 to the nsp12, and the binding affinity constant was shown. Purified CDK2 protein was diluted to different concentrations (125, 250, 1000, and 2000 nM), and then nsp12 proteins (50 μg/mL) was biotinylated and captured on streptavidin (SA) biosensors. The association and dissociation curves of CDK2 proteins are shown. **e** 6*His-tagg**e**d nsp12 was pulled down by GST-tagged CDK2 in vitro, then nsp12 and CDK2 proteins were analyzed with indicated antibodies 6*His or GST

We next performed co-immunoprecipitation and Western blot to validate and further examine the interaction between CDK1 and CDK2 with the RdRp complex by transfecting cells to express either nsp7, nsp8 or nsp12 together or individually. The results showed specific association of CDK2 with the RdRp complex, also with nsp12 itself, and CDK2 association was much weaker for nsp8 and not detectable for nsp7 (Fig. 2a). Compared with CDK2, very little CDK1 was detected in the immunoprecipitated materials, and no CDK5 was detected (Fig. 2a). To further examine the specific interaction of CDK2 with nsp12, we performed the proximity ligation assay (PLA) which displays protein-protein interactions in situ with individual fluorescent dots. As shown in Fig. 2b, the most red fluorescent dots were observed in nsp12-expressing cells, less in cells expressing nsp8, and very few in nsp7-expressing cells, which are consistent with the Co-IP data (Fig. 2a). The number of PLA red fluorescent dots were counted and the PLA results revealed a 28 and 2.7-fold increase in the association of CDK2 with nsp12 over nsp7 and nsp8, respectively (Fig. 2c). Fewer interaction foci were observed with RdRp compared with nsp12 alone (Fig. 2c), which may have resulted from relatively lower expression level of nsp12 in cells co-transfected with nsp7, nsp8 and nsp12 than in cells only expressing nsp12 (Fig. 2a). Together, these data demonstrate the interaction between CDK2 and nsp12, which allows CDK2 to promote RdRp activity shown in Fig. 1.

To test if CDK2 binds to nsp12 directly, we therefore measured the binding affinity between CDK2 and nsp12 by biolayer interferometry assay (BLI assay), which is extensively used for the analysis and characterization of protein interactions. CDK2 exhibited a binding affinity to nsp12 in a concentration-dependent manner, with an equilibrium dissociation constant (KD) of 260 ± 41 nM (Fig. 2d). In line with the BLI result, CDK2 was showed to directly bind to nsp12 in an in vitro GST pull-down assay (Fig. 2e). Taken together, these results suggest a direct binding of nsp12 to CDK2.

### CDK2 enhances the RNA synthesis by SARS-CoV-2 RdRp

**Fig. 3:**
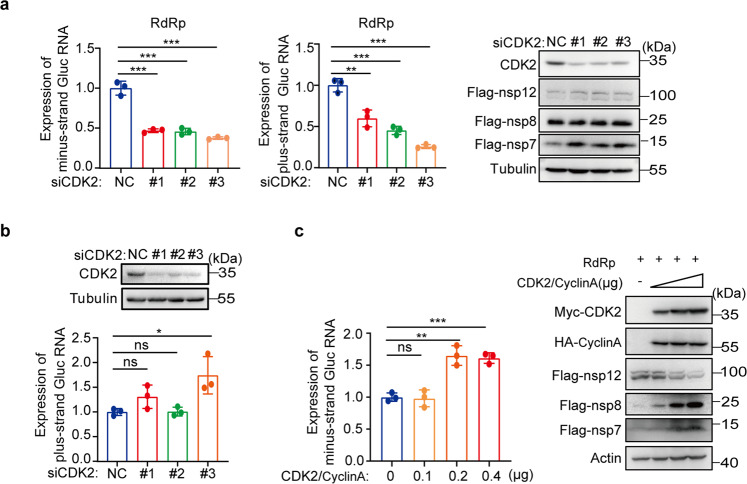
CDK2 knockdown specifically impairs the activity of RdRp. **a**, **b** HEK293T cells expressing CoV-Gluc, nsp12, nsp7, nsp8 plasmid DNA at the ratio of 1:10:30:30 (**a**) or control vector and CoV-Gluc (**b**) were transfected with three siRNA specific sequence for CDK2. After 48 h, minus-strand or plus-strand Gluc-RNA was detected by qRT-PCR and protein expression was detected by Western blot analysis. **c** CoV-Gluc, nsp12, nsp7, nsp8 plasmid DNA were co-transfected in HEK293T cells at the ratio described above, and then a series of CDK2/CyclinA concentration plasmids were transfected into six-well plates. After 48 h, minus-strand Gluc-RNA was detected by qRT-PCR and protein expression was detected by western blot analysis. Data are presented as mean ± SD, *P < 0.05, **P < 0.01, ***P < 0.001 and ns not significant (two-tailed unpaired Student’s *t*-test)

We next assessed the effect of CDK2 knockdown on the levels of plus-strand and minus-strand RNA in the CoV-RdRp-Gluc reporter assay by performing RT-qPCR. Results show that knockdown of CDK2 by either of three siRNAs reduced the levels of both the minus- and plus-strand Gluc-RNA by 40–75%, without measurable effect on the expression of either nsp7, nsp8, or nsp12 (Fig. 3a). In the meantime, CDK2 knockdown did not affect the expression of plus-strand Gluc-RNA in the control cells that did not express viral RdRp (Fig. 3b). The same findings were obtained from HeLa cells (Supplementary Fig. 2a, b). In addition, we found that the level of minus-strand RNA in the CoV-RdRp-Gluc system increased significantly accompanied with increasing level of CDK2 (Fig. 3c). These results support a role of CDK2 in assisting SARS-CoV-2 RdRp-mediated RNA expression (Fig. 1).

### Nsp12 is phosphorylated by CDK2 for efficient RNA synthesis

**Fig. 4:**
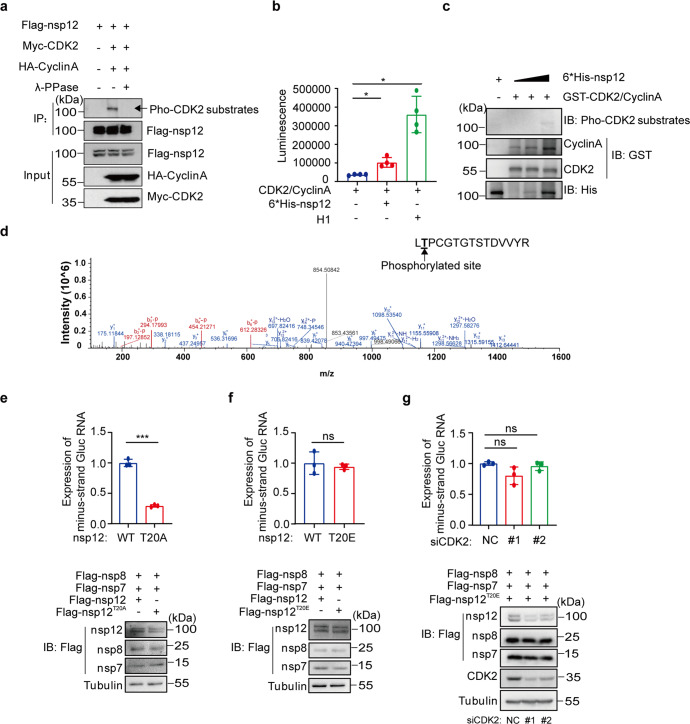
Phosphorylation of nsp12 by CDK2 enhances the RdRp activity. **a** Flag-nsp12, Myc-CDK2, and HA-CyclinA were co-transfected in HEK293T cells for 48 h. Flag-tagged nsp12 protein was immunoprecipitated according to the previous experimental method and then treated with/without λ-PPase, followed by immunoblotting with antibodies against Pho-CDK2 substrates, Flag, HA, and Myc. **b** CDK2/CyclinA Kinase Enzyme Systems with the ADP-Glo^TM^ Assay. Purified nsp12 or Histone H1 incubated in a kinase reaction mixture containing 50 μM ATP and 6.4 ng active CDK2/CyclinA. The experiments were performed in quadruplicate. Data are shown as mean ± SD, two-sided Mann–Whitney test, *p < 0.05. **c** In vitro CDK2/CyclinA kinase assay. Purified CDK2/CyclinA was incubated with recombinant proteins nsp12 in vitro phosphorylation system. The reaction mixture was collected and examined using immunoblotting. **d** Phosphorylation site in nsp12 was identified by mass spectrometry (MS). **e**, **f** CoV-Gluc, Flag-nsp7, Flag-nsp8, Flag-nsp12, or T20 mutant nsp12 were co-transfected in HEK293T cells for 48 h. The minus-strand Gluc-RNA was quantified by RT-qPCR. Cell lysates were analyzed by immunoblot with the indicated antibodies. **g** HEK293T cells were transfected with Flag-nsp7, Flag-nsp8, Flag-nsp12 mutant T20E, Gluc-RNA plasmids and individual siRNAs for 48 h. The minus-strand Gluc-RNA was quantified by RT-qPCR and protein expression was detected by Western blot analysis. The experiment was performed in triplicate. Data are shown as mean ± SD, two-tailed unpaired Student’s *t*-test in **e**–**g**, ***P < 0.001, ns not significant

Given the kinase activity of CDK2, the association of CDK2 with nsp12 may lead to phosphorylation of nsp12 or other components of viral RdRp complex, thus affecting the activity of RdRp. To test this, we used an active CyclinA-CDK2 complex approach^27^ in which the substrate of CDK2 is phosphorylated by the active CyclinA-CDK2 complex and the reaction is inhibited by lambda protein phosphatase (λ-PPase). Plasmids expressing CDK2, CyclinA, either of nsp7, and nsp8, and nsp12 were co-transfected into HEK293T cells, followed by treating the cell lysate with λ-PPase. The result revealed a band of nsp12, which was lost upon the treatment with λ-PPase (Fig. 4a), whereas no band was observed from the cell lysate expressing either nsp7 or nsp8 (Supplementary Fig. 3a, b), suggesting only nsp12 acts as a substrate for CDK2.

To further verify nsp12 can be phosphorylated by CDK2, we first in vitro assessed the level of nsp12 phosphorylation in the presence of CDK2 in complex with CyclinA2, determined by ADP-Glo^TM^ Kinase Assay kit. The assay measures the luminescent signal of ADP produced from a kinase reaction, which positively correlates with kinase activity. The results showed that the addition of purified nsp12 to a kinase reaction mixture resulted in a strong luminescence signal (Fig. 4b), suggesting a kinase reaction occurred. In addition, another in vitro kinase assay was performed using purified CDK2, Cycliln A, and nsp12 in kinase reaction buffer, resulting in a protein band reacted with antibodies specific for Pho-CDK2 (Fig. 4c). Taken together, our results strongly suggest that nsp12 is a substrate of CDK2.

To validate this result, we determined the phosphorylation site of nsp12 through liquid chromatography-mass spectrometry analysis of immunoprecipitated nsp12. The data revealed a CDK family phosphorylation site at T20 in nsp12 (Fig. 4d), no such phosphorylation site was identified for nsp7 and nsp8 (data not shown). Together, our results strongly suggest that nsp12 is phosphorylated at amino acid T20 by CDK2.

To reveal the functional relevance of nsp12 phosphorylation at T20, we constructed two nsp12 mutants, the phosphor-mimetic T20E and the non-phosphorylated T20A, and examined their ability to support viral RNA synthesis in the CoV-RdRp-Gluc reporter assay. The result showed that with a similar expression level of the wild-type or the mutated nsp12, the T20A mutation reduced the level of the minus-strand RNA by more than 60% compared with that of the wild-type nsp12 (Fig. 4e), and the phosphor-mimetic nsp12 mutant T20E was as efficient as the wild-type nsp12 in RNA expression (Fig. 4f). This suggests the importance of the nsp12 phosphorylation at T20 in supporting the efficient viral RNA synthesis. Importantly, we observed that the minus-strand RNA synthesis by the phosphor-mimetic T20E or T20A was not affected by CDK2 knockdown (Fig. 4g, Supplementary Fig. 3c), supporting the mechanism of CDK2 stimulating RdRp function through phosphorylating T20 in nsp12. Taken together, these data suggest that nsp12 is phosphorylated by CDK2 and the phosphorylation of nsp12 by CDK2 enhances the RdRp activity.

### CDK2 promotes the formation of the RdRp complex

**Fig. 5:**
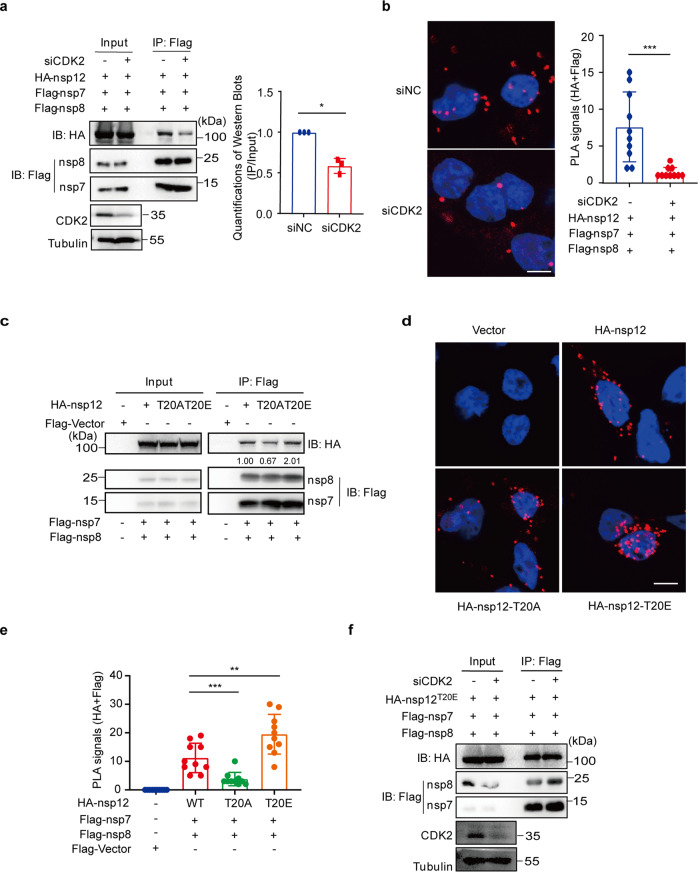
CDK2 stabilizes the RdRP complex. **a** HEK293T cells were transfected with control siRNA or CDK2 siRNA and then transfected with vectors expressing Flag-tagged nsp7/8 and HA-tagged nsp12. Cell lysates were immunoprecipitated according to the previous experimental method. The experiments were performed in triplicate and western blots were quantified using ImageJ. Data are shown as mean ± SD, *P < 0.05 (two-tailed unpaired Student’s *t*-test). **b** Situ proximity ligation assay (PLA) and confocal imaging to show the interaction of HA-nsp12 with Flag-nsp7, Flag-nsp8 (red dots) in HEK293T cells which were transfected with control siRNA or CDK2 siRNA. Nuclei were stained with DAPI (blue). Scale bars, 5 μm. Number of PLA red fluorescent dots in randomly selected cells, *n* = 10 cells per group. Data are shown as mean ± SD, two-sided Mann–Whitney test, ***P < 0.001. **c** Flag-tagged nsp7/8 and HA-tagged nsp12 or various nsp12 mutants were co-transfected in HEK293T cells for 48 h. Anti-Flag M2 affinity gel was used for Co-Immunoprecipitation and Western blot analysis was performed with indicated antibodies. **d** Situ proximity ligation assay and confocal imaging to show the interaction of HA-tagged nsp12 or nsp12 mutants with Flag-nsp7, Flag-nsp8 (red dots) in HEK293T cells. Representative data are shown. Nuclei were stained with DAPI (blue). Scale bars, 5 μm. **e** Number of PLA red fluorescent dots in randomly selected cells, *n* = 10 cells per group. Data are shown as mean ± SD, two-tailed unpaired Student’s *t*-test between group T20E and group WT (wide type), two-sided Mann–Whitney test between group T20A and group WT, **P < 0.01, ***P < 0.001. **f** HEK293T cells were transfected with control siRNA or CDK2 siRNA, and then transfected with vectors expressing Flag-tagged nsp7/8 and HA-tagged nsp12 T20E mutant plasmid DNA. Anti-Flag M2 affinity gel was used for Co-Immunoprecipitation and Western blot analysis was performed with antibodies against HA, Flag, CDK2, or tubulin

Next, we investigated how CDK2-mediated phosphorylation of nsp12 at T20 affects the viral RNA synthesis. First, we knocked down CDK2 and examined the effect on nsp12 expression. None of the three different pairs of CDK2 siRNA altered the level of nsp12, suggesting that CDK2-induced phosphorylation of nsp12 at T20 does not affect nsp12 expression (Supplementary Fig. 4). We further assessed the possible effect of CDK2 silencing on the formation of the RdRp complex in a co-immunoprecipitation assay. The RdRp complex in cell lysates was pulled down using the anti-Flag-M2 beads targeting nsp7/8-Flag, followed by western blotting to detect HA-nsp12 using the HA antibody. The results showed a 1.7-fold decrease in the amount of HA-nsp12 that was co-precipitated with nsp7/8-Flag when CDK2 was knocked down (Fig. 5a). In support of the Co-IP data, the results of PLA assay revealed a marked reduction in the interaction between nsp12 and either nsp7 or nsp8 with CDK2 knockdown (Fig. 5b). Together, these data suggest an important role of CDK2 in the formation of the RdRp complex in cells.

To determine whether CDK2 enhances the RdRp complex formation through phosphorylation of T20 in nsp12, we examined the T20A and T20E nsp12 mutants in the Co-IP and PLA assays as described above. The results showed that with a similar expression level of wild-type or mutated nsp12, the T20A mutation caused a 33% decrease in the amount of nsp7/8/12 RdRp complex compared to the wild-type, while the phosphor-mimetic T20E mutation caused a two-fold increase in the precipitated nsp7/8/12 complex (Fig. 5c). In support of the Co-IP data, results of PLA showed markedly decreased interaction of T20A nsp12 with nsp7/8 and increased interaction of T20E nsp12 mutant with nsp7/8 (Fig. 5d, e). Further, we noted that CDK2 knockdown did not affect the amount of T20E nsp12 in the nsp7/8 immunoprecipitates (Fig. 5f), consistent with the independence of this nsp12 mutant on CDK2 in catalyzing RNA synthesis (Fig. 4f), suggesting that the role of CDK2 in the formation and the function of the RdRp complex is to phosphorylate the T20 amino acid of nsp12.

### CDK2 inhibitor SNS-032 blocks SARS-CoV-2 replication

**Fig. 6:**
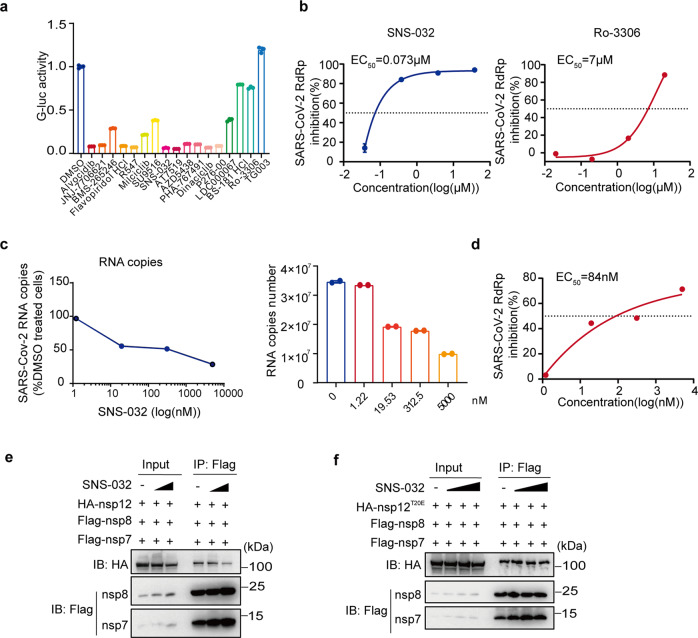
CDK2 inhibitor SNS-032 blocks SARS-CoV-2 replication. **a** CoV-Gluc, nsp12, nsp7, and nsp8 plasmids were co-transfected at the ratio of 1:10:30:30 in HEK293T cells. Twelve hours post-transfection, different compounds of CDKs family inhibitors (10 μM) were added to the cells. Gluc activity was tested after 24 h. The tests were performed in three independent experiments, data are shown as mean ± SD. **b** CoV-Gluc, nsp12, nsp7, and nsp8 plasmid DNA were co-transfected in HEK293T cells at the ratio described above. Serially diluted inhibitors SNS-032 and Ro-3306 were added into HEK293T cells after 12 h. Gluc activity was measured after 24 h of incubation. The experiments were performed in triplicate. **c** Vero cells were treated with 1.22, 19.53, 312.5, 5000 nM SNS-032 and then infected by SARS-COV-2 at MOI = 0.05. Viral infection was determined using qPCR. Experiments were performed two times. Error bars indicate mean ± SD. **d** EC_50_ of SNS-032 was calculated. **e**, **f** Flag-tagged nsp7/8 and HA-tagged nsp12 (**e**) or HA-tagged nsp12 T20E mutant (**f**) plasmid DNA were co-transfected in HEK293T cells. 12 h post-transfection, serially diluted inhibitors SNS-032 (**e**: 0, 0.039, 0.6 μM; **f**: 0, 0.039, 0.15, 0.6 μM) were added into HEK293T cells. After 24 h incubation, anti-Flag M2 affinity gel was used for Co-Immunoprecipitation and western blot analysis was performed with antibodies against HA, Flag

The enhancing effect of CDK2 on viral RNA synthesis prompts us to test whether CDK2 inhibitors can inhibit SARS-CoV-2 infection. To this end, we assessed a total of 17 CDKs inhibitors in the CoV-Gluc reporter assay. The drug target description targets for each inhibitor can be found in Supplementary Table S3. The first 13 inhibitors either are specific for CDK2 or possess a broad activity against multiple CDKs including CDK2, and the rest three are specific for CDK1, CDK7, CDK9, and CIK. Compared with the DMSO control group, all CDK2 inhibitors potently inhibited the Gluc activity, with 10 of 13 causing more than 60% inhibition, whereas neither of other CDKs inhibitors showed any inhibitory effect (Fig. 6a), suggesting the specificity of CDK2 inhibitors in inhibiting SARS-CoV-2 RdRp activity. In further support of this conclusion, the CDK2 inhibitor SNS-032 inhibited the activity of SARS-CoV-2 RdRp with an EC_50_ of 73 nM (Fig. 6b), as opposed to the CDK1 inhibitor Ro-3306 exhibiting no significant effect on Gluc expression up to 7 μM (Fig. 6b), which is more than 600-fold higher than the reported IC_50_ 110 nM against CDK1.^28^ Finally, we evaluated the antiviral activity of SNS-032 against SARS-CoV-2 (MOI of 0.05) by infecting Vero cells and measuring viral infection by quantifying viral genomic RNA with RT-qPCR. As shown in Fig. 6c, infection of live SARS-CoV-2 on Vero cells was also strongly inhibited by SNS-032 treatment. SNS-032 markedly inhibited SARS-CoV-2 infection in a dose-dependent manner, with an EC_50_ of 84 nM (Fig. 6d). Together, these results identify CDK2 as a potential target of anti-SARS-CoV-2 drugs.

To explore the mechanics of how SNS-032 inhibits SARS-CoV-2 replication, we assessed the effect of SNS-032 on the formation of the RdRp complex in a Co-IP assay. The results showed a significant decrease in the amount of HA-nsp12 in the presence of SNS-032, suggesting an inhibitory effect of SNS-032 on the formation of RdRp complex (Fig. 6e). In contrast, upon replacing wide type nsp12 with phosphor-mimetic T20E mutant, the Co-IP assay showed no effect of SNS-032 on the formation of the complex (Fig. 6f). Together, our results suggest that SNS-032 targets the CDK2 and inhibits the formation of the RdRp complex by blocking phosphorylating the T20 amino acid of nsp12.

## Discussion

In this study, we have identified CDK2 as a host factor associating with SARS-CoV-2 RdRp and promoting viral RNA replication. Specifically, our data showed that CDK2 interacts with nsp8 and nsp12 (Fig. 2), is required for efficient viral RNA synthesis (Fig. 3), phosphorylates nsp12 at amino acid T20 (Fig. 4), and promotes the formation of the RdRp complex (Fig. 5). We further showed that CDK2-mediated phosphorylation of nsp12 at T20 is required for both the RdRp activity and the RdRp complex assembly. Taken together, our data support SARS-CoV-2 hijacking CDK2 to phosphorylate nsp12 which in turn assists the assembly of the RdRp complex and ensures efficient synthesis of viral RNA.

As an enzyme essential for the viral RNA replication and thus the important antiviral target,^29,30^ RdRps of many RNA viruses have been intensively studied; yet posttranslational modifications of RdRp and their roles in viral RNA replication and infection are under studied.^31^ One study reported phosphorylation of hepatitis C virus (HCV) RdRp by protein kinase C-related kinase 2 and its role in supporting HCV RNA replication.^32^ Here, we present another example that SARS-CoV-2 nsp12, the RdRp enzyme, is phosphorylated by cellular kinase CDK2 at T20, which is required for both the RdRp function and the RdRp complex assembly. It is pivotal to test whether the RdRps of other coronaviruses are also the substrates of CDK2 and whether their RNA synthesis function is dependent on CDK2-mediated phosphorylation, which may lead to the development of pan-coronavirus antivirals by targeting CDK2.

The LC-MS analysis reveals T20 as the sole phosphorylation site of nsp12 (Fig. 4c). Importance of T20 phosphorylation in nsp12 function is supported by the impaired RNA synthesis activity of the T20A mutant and the wild-type activity of the phosphor-mimetic mutant T20E. Since the GPS2.1 software also predicts T226 and T926 as potential sites being phosphorylated by CDK2, we tested the T226A and T926A mutants which showed moderate reduction in viral RNA synthesis (Supplementary Fig. 3d), while the phosphor-mimetic T226E or T926E mutant displayed wild-type level activity (Supplementary Fig. 3e). It is possible that T226 and T926 serve as minor or secondary phosphorylation sites by CDK2 and also contribute to nsp12 RNA synthesis function. Specific pT20, pT226, and p926 antibodies will help to verify their phosphorylation in the context of nsp12 and their potentially different roles in RdRp function. In addition, we analyzed the amino acids sequence of nsp12 among SARS-CoV-2 VOCs including Alpha (B.1.1.7), Beta (B.1.351), gamma (P.1), Delta (B.1.617.2), and identified only four different amino acids. Currently, the possibility cannot be excluded that these mutations in nsp12 may affect its interaction with CDK2. However, T20 was found highly conserved among different strains, suggesting that the CDK2-mediated phosphorylation of T20 might be shared among SARS-CoV-2 variants.

It is unclear how T20 phosphorylation enhances the interaction of nsp12 with nsp7/8. Structural study suggests that a β-hairpin of nsp12 which consists of D29 to K50 at the N-terminus inserts into the groove formed by the NiRAN and the palm, resulting in several close interaction that stabilize the overall RdRp structure.^9^ T20 is located upstream of the β-hairpin, and the function of this flexible 20 amino acids N-terminal region remains largely unknown. T20 phosphorylation may promote the formation of the RdRp complex either by enhancing the interaction of nsp12 with nsp7/8 or stabilizing the RdRp complex.

Several viruses have been reported to target cellular CDKs, thus disrupting cell cycle progression and/or overcoming cellular constraints of virus infection.^23^ It has also been shown that SARS-CoV-2 infections may lead to reduce CDK1/2 activity, resulting in an S/G2 arrest and likely creating cellular conditions favoring viral replication.^33^ Further, it has been proposed that the N protein of coronavirus SARS-CoV is highly conserved among these viruses and may interact with cyclinD, resulting in the inhibition of the CDK activity.^19^ We herein showed the association of the RdRp with CDK1 and CDK2. CDK1 or CDK2 expression would affect RdRp function, though the CDK1 inhibitor has little effect on the viral replication. Given that CDK1 is believed to play a significant role in G2/M phase and CDK2 is reported in late S phase to promote the mitosis, it is possible that CDK1 and CDK2 may play different roles in regulating viral replication at different phases of the cell cycle. Beside the function of the interaction to warrant viral RNA synthesis, this may also contribute to modulate the cell cycle for viral infection.

CDK inhibitors have been pursued as cancer treatments, and evaluated in clinical trials. Among the 17 CDK inhibitors we tested against SARS-CoV-2 RdRp activity, all 14 inhibitors of CDK2 showed significant inhibitory activity. Particularly, CDK2 inhibitor SNS-032 showed the strongest inhibition of SARS-CoV-2 RdRp and markedly inhibits SARS-CoV-2 infection. SNS-032 (formerly BMS-387032) was originally discovered as a selective inhibitor against CDK2 by Bristol-Myers Squibb Pharmaceutical Research Institute (Stamford, CT).^34^ Subsequent studies revealed that SNS-032 inhibited the proliferation of tumor cells by interfering with CDK7 and CDK9 with IC_50_ of 62 and 4 nM respectively.^11,32,35^ More studies are needed to determine whether the strong anti-SARS-CoV-2 activity of SNS-032 also depends on the inhibition of CDK7 and/or CDK9. The CC_50_ value of SNS-032 reported previously is approximate 14 to 70 folds of EC_50_,^36^ which is consistent with our data showing a CC_50_ value of 87 μM in Vero cells (Supplementary Fig. 5), suggesting an acceptable selective index of SNS-032 for the treatment of SARS-CoV-2 infection.

Together, our study uncovers a new mechanism regulating the function of SARS-CoV-2 RdRp through viral hijacking cellular kinase CDK2 to phosphorylate RdRp at amino acid T20. With the possibility that other coronaviruses may also depend on CDK2 or other CDKs for efficient viral RNA replication, CDK inhibitors hold the promise of being repurposed as pan-coronaviruses antivirals.

## Materials and methods

### Cells, viruses, and compounds

HEK293T cells, HeLa cells and Vero cells were obtained from ATCC and cultured in DMEM (Gibco) containing 10% FBS (Gibco). The cells were grown in a 37 °C incubator with 5% CO_2_. The SARS-CoV-2 virus were collected from confirmed COVID-19 patients as previously described.^37–39^ All the SARS-CoV-2 relevant experiments were performed in the biosafety level 3 (BSL-3) laboratory which is supported by the Institute of Pathogen Biology, Chinese Academy of Medical Sciences and Peking Union Medical College (Beijing, China). All compounds used in Fig. 6a for screening were from Selleck chemicals (Houston, TX, USA). SNS-032 (T6049) and Ro-3306 (T2356) used in the Fig. 6b, d and Fig. S5 were purchased from Target Mol (Boston, MA, USA) and dissolved in DMSO. All compounds have a purity of 95%.

### Plasmids and reagents

Flag-nsp12, Flag-nsp7, Flag -nsp8 were constructed into the pCMV6-Entry vector as described in detail previously.^26^ Nsp12 mutant constructs, containing the combined T20A and T20E mutation were obtained using Fast Mutagenesis System (TransGen Biotech). Myc-CDK2 was obtained from Origene (Cat# RC200494) and pLVX-CCNA2-HA-IRES-Puro was purchased from ZOMANBIO Co., Ltd (Cat# ZK7080). Human Flag-CDK1 and EGFP-CyclinB were cloned into the pSG5 vector and EGFP-N1 vector in our lab. Full-length GST-CDK2 and GST-CyclinA were cloned into pGEX-4T1 vector in our lab. Plasmids were transfected to the HEK293T cells or HeLa cells using Vigofect (Vigorous) or lipo2000 (Invitrogen) transfection reagents according to the user’s manual.

### Western blot and antibodies

Antibodies used in western blot analysis are rabbit anti-Flag tag (CST, 14793 S), rabbit anti-HA tag (CST, 3724 S), rabbit anti-CDK2 (CST, 18048 S), rabbit anti-CDK2 (Proteintech, 10122-1-AP), mouse anti-Tubulin (Sigma, T5168), mouse anti-Myc tag (CST, 2276 S), rabbit anti-CDK1 (Proteintech, 19532-1-AP), rabbit anti-CDK5 (Proteintech, 10430-1-AP), rabbit beta-Actin (CST, 8457 T), mouse anti-Flag tag (Sigma, F3165), mouse anti-6*his Tag (BBI, D191001), rabbit anti-GST Tag (Proteintech, 10000-0-AP). Western blot analyses were performed as previously described.^40^ The cells were collected after washed with PBS (PH 7.4), and lysed using NP-40 buffer (Beyotime, P0013F) with protease inhibitor cocktail (Roche). Briefly, protein lysates samples were electrophoresed on 10% or 12% SDS-polyacrylamide gels, and transferred to 0.45 µm PVDF membrane (Millipore). Membranes were blocked with 5 % non-fat milk supplemented in 0.1% TBST. After washed with TBST, primary antibodies was incubated with PVDF membrane overnight at 4 °C. Then the blots were incubated with HRP-conjugated antibodies (1: 5000) which obtained from Jackson Immuno Research for 1 h. Images were acquired on ChemiDoc^TM^ MP Imaging System (BIO-RAD).

### Immunoprecipitation

HEK293T cells were lysed using IP buffer (Beyotime, P0013F). Cell lysates were incubated on Rotating Mixer for 30 min at 4 °C, and then centrifuged (12,000 × *g*) for 10 min in a low temperature high speed centrifuge. The supernatants were incubated with Flag M2 affinity gel (Sigma, A2220) at 4 °C overnight. The Flag M2 affinity gel were washed with IP buffer four times. The immunoprecipitates were analyzed by Western blot.

### Mass spectrometry

For the nsp12/7/8 interacting proteins identification, cells were lysed using IP buffer (Beyotime, P0013F) after 48 h. The RdRp complexes were pulled down with Flag M2 affinity gel (Sigma, A2220), and separated on SDS-PAGE. Proteins were directly digested with trypsin and the peptides were dried for LC-MS analysis. For the phosphorylated peptides of nsp12 identification, HEK293T cells were transduced with Myc-CDK2, HA-CyclinA, Flag-nsp12 or control vector. Cells were lysed using IP buffer (Beyotime, P0013F) supplemented with phosphatase inhibitors (Roche) after 48 h. Flag-nsp12 were pulled down with Flag M2 affinity gel and loaded for SDS-PAGE. The peptides were subjected to LC-MS analysis And the MS/MS data were analyzed using Proteome Discoverer 2.1.

### RNA interference

CDK2 specific siRNA was purchased from JTSBIO Co., Ltd (Wuhan, China), with target sequences, #1 5’-CCAUCAAGCUAGCAGACUUTT-3’, #2 5’-CCAGCUCUUCCGGAUCUUUTT-3’, and #3 5’-CCUCCACCGAGACCUUAAATT-3’. Control siRNA target sequence 5’-UUCUCCGAACGUGUCACGUTT-3’. Cells were transfected siRNA by using LipoRNAiMAX (Invitrogen) transfection reagent and fresh complete medium was added after 12 h.

### Gluc activity assay

SARS-CoV-2 polymerase activity assay system used in this study as previously described.^26,37^ Briefly, the coelenterazine-h (Promega) was dissolved into 1.022 mmol/L with absolute ethyl alcohol. Before Gluc activity assay, the coelenterazine-h was diluted 60 times in PBS and incubated 30 min at room temperature in the dark. Supernatant (10 μL) was added to a white and opaque 96-well plate and coelenterazine-h (60 μL) was injected to each well. The supernatant was followed by luminescence analysis (integration time 0.5 s) using Berthold LB 960 microplate luminometer (Berthold Technologies).

### In situ proximity ligation assay (PLA)

The Duolink proximity ligation assay (PLA) was performed according to the manufacturer’s protocol (Sigma–Aldrich). HEK293T cell (4 × 10^5^) were seeded in 20 mm glass bottom dishes, and then 1 μg Flag-nsp12 or Flag-nsp7 or Flag-nsp8 were transfected using Vigofect. Cells were fixed with 4% paraformaldehyde after 48 h and permeabilized with 0.02% Triton X-100. Samples were blocked with blocking solution for 1 h and then stained with both anti-CDK2 (Proteintech, 10122-1-AP, 1:100) and mouse anti-Flag antibodies (Sigma, F3165, 1:5000) together for 1 h. Next, cells were incubated with secondary antibodies which are conjugated with Duolink PLA minus and plus oligonucleotide probes for 1 h. Then, the samples were incubated with ligase buffer for 30 min and further incubated with the amplification solution which can amplify the signal of ligated PLA probes for 100 min. The above reactions were carried out at 37 °C. Samples were washed with wash buffer B three times. Nuclei were stained with DAPI (blue). Images were acquired with a ×100 Oil objective on Perkin Elmer Ultra View VoX confocal (Perkin Elmer).

### Biolayer interferometry (BLI) binding assay

We used ForteBio Octet RED96 instrument (ForteBio, Inc., CA, USA) to analyze the proteins interactions. The purified 6*His-nsp12 (50 μg/mL) protein was biotinylated with EZ-Link NHS-Biotin Reagents (Thermo, #21343) at room temperature and then captured by Streptavidin biosensors (ForteBio Inc., Menlo Park, CA). Ligand biosensors and reference biosensors were immersed in a different concentration gradients of CDK2 proteins for association 180 s, then dissociation 180 s. A blank binding buffer with no proteins was used to correct the baseline. The binding affinity constant KD was calculated using Data Analysis 9.0 software on the ForteBio Octet RED instrument.

### GST pull-down assay

1.5 μg of 6*His-nsp12 were co-incubated with GST-immobilized CDK2 in 600 μL pull-down buffer which containing 150 mM NaCl, 50 mM Tris-HCI pH 7.5, 5 mM DTT, 0.1% NP-40, 0.25 mg/mL BSA overnight at 4 °C with rotation.^41^ Beads were washed for five times with no BSA pull-down buffer. Beads were boiled with SDS loading buffer and boiled 10 min. The samples were examined by Western blot.

### Luminescence analysis for in vitro phosphorylation

0.3 μg 6*His-nsp12 recombinant proteins (CUSABIO BIOTECH CO., LTD. https://www.cusabio.com/) and 6.4 ng CDK2/CyclinA enzyme and 50 μM ATP were incubated in kinase reaction buffer from ADP-Glo^TM^ Kinase Assay kit (Promega) for 10 min. Then, add ADP-Glo^TM^ Reagent and incubate for 40 min. Next, Kinase Detection Reagent was added to the mixture for 30 min. The supernatant was followed by luminescence analysis (integration time 0.5 s).

### CDK2 kinase assay

60 ng GST-CDK2 and GST-CyclinA were incubated together for 12 h in advance and then incubated with 6*His-nsp12 (0.33 μg or 1 μg or 3 μg) in 30 μL kinase buffer (50 mM Tris-HCl (pH 7.5), 1 mM EGTA, 10 mM MgCl_2_, 2 mM DTT, 50 mM cold ATP). The reactions were maintained at 30 °C for 30 min,^42^ and then quenched with SDS loading buffer and examined by Western blot.

### RNA isolation and real-time PCR

Total RNA was isolated using RaPure Total RNA Kit (Magen, R4011-03). Then, RNA was used to perform the reverse transcription for each sample with primer for 1 h at 37 °C. The minus-Gluc-RT primer sequence is 5′-ACTGTCGTTGACAGGACACG-3′ and plus-Gluc-RT primer sequence is 5′-TGGATCTTGCTGGCGAATGT-3′. Real-time quantitative PCR was carried out with the SYBR Green Master Mix (Applied Biosystems, A25742) on an ABI StepOnePlus^TM^ system. Levels of mRNA expression were calculated and levels of actin mRNA was used for normalization. The primers used in real-time PCR analysis were synthesized by Biomed Beijing and their sequences are presented in supplementary Table S2.

### SARS-CoV-2 infection assay

Vero cells (1 × 10^4^) were cultured overnight in 96-well plates. Different concentrations of the compounds were added to the cells for one hour before SARS-CoV-2 was added. Then, SARS-CoV-2 (MOI of 0.05) was added and incubated for 1 h. Viral medium was removed and replaced with fresh complete medium containing drug and incubated for 24 h. Viral RNA was separated from the supernatants using the Direct-zol RNA mini Prep kit (Zymo research) and its level was determined with the TaqMan Fast Virus 1-step Master Mix (Applied Biosystems). The primer pair used to detect the viral RNA target SARS-CoV-2 nucleocapsid gene. The primer sequences specific for SARS-CoV-2 nucleocapsid gene are F: 5′-AACACAAGCTTTCGGCAGAC-3′ and R: 5′-AGCTGTGTAGGTCAACCACG-3′. The probe sequence is 5′-CAGCGCTTCAGCGTTCTTCGGAATGTCGC-3′. The standards were diluted from 1 × 10^9^ copies to 1 × 10^3^ copies every tenfold and the copy number of SARS-CoV-2 virus RNA was calculated based on the standard curve. We used the GraphPad Prism8 software to fit the dose-response curves.

### Cell toxicity assay

Vero cells were cultured overnight at a density of 1 × 10^4^ cells/well in 96-well plates and then SNS-032 was added at the indicated concentration for 24 h. CCK-8 was added and incubated at 37 °C for 1 h and then supernatant was measured according to Manufacturer’s instructions of Cell Counting kit-8 (Meilunbio).

### Statistical analysis

All grouped data are reported as mean ± SD. Differences between the groups were compared using unpaired Student’s *t*-tests or the two-sided Mann–Whitney U test when anormal distribution could not be assumed. Graphpad Prism 8 was used for all statistical analyses.

## Supplementary information

Supplementary-Materials

## Data Availability

The mass spectrometry proteomics data have been deposited to the ProteomeXchange Consortium (http://proteomecentral.proteomexchange.org) via the iProX partner repository^43^ with the dataset identifier PXD037068. The other datasets generated in this study are available from the corresponding author upon reasonable request.
