# Supplementary material for: SARS-CoV-2 hijacks cellular kinase CDK2 to promote viral RNA synthesis
Source: Signal Transduct Target Ther. 2022 Dec 27;7:400. doi: 10.1038/s41392-022-01239-w (PMC9793359; doi:10.1038/s41392-022-01239-w)
Supplement: Supplementary file 1 — Supplementary-Materials [file 41392_2022_1239_MOESM1_ESM.docx]

Supplementary Materials for

SARS-CoV-2 hijacks cellular kinase CDK2 to promote viral RNA synthesis

Saisai Guo^1#^, Xiaobo Lei^2#^, Yan Chang^3#^, Jianyuan Zhao^1#^, Jing Wang^1#^, Xiaojing Dong^2^, Qian Liu^1^, Zixiong Zhang^1^, Lidan Wang^1^, Dongrong Yi^1^, Ling Ma^1^, Quanjie Li^1^, Yongxin Zhang^1^, Jiwei Ding^1^, Chen Liang^4^, Xiaoyu Li^1^, Fei Guo^2*^, Jianwei Wang^2*^, Shan Cen^1,5*^.

Correspondence to:

shancen@imb.pumc.edu.cn (S.C.), wangjw28@163.com (J.W.), guoafei@ipbcams.ac.cn (F.G.)

**This PDF file includes:**

Figures. S1 to S5

Tables S1 to S3


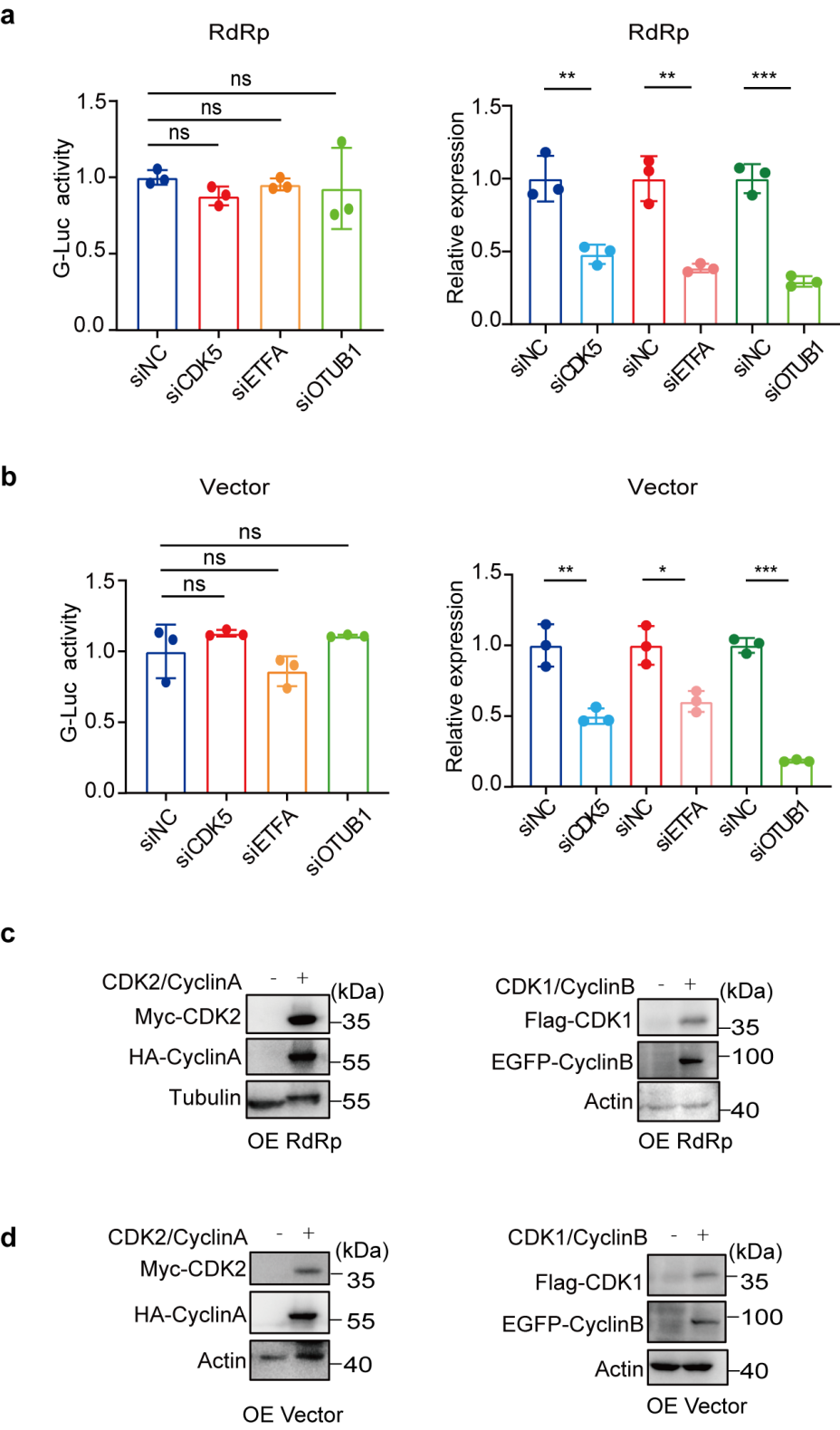


**Figure. S1. The effect of candidate genes knockdown on the function of SARS-CoV-2 RdRp.** **a, b** HEK293T cells expressing Flag-RdRp (**a**) or control vector (**b**) were transfected with CoV-Gluc, and then either CDK5, ETFA or OTUB1 siRNA (three siRNAs per gene) were transfected for 48 hours. Then the Gluc activity was measured. Candidate gene knockdown was determined by qRT-PCR. Experiments was performed in triplicate, data are presented as mean ± SD, *P < 0.05, **P < 0.01, ***P < 0.001 and ns, not significant (two-tailed unpaired Student’s t-test). **c, d** HEK293T cells expressing CoV-Gluc, nsp12, nsp7, nsp8 plasmid DNA at the ratio of 1:10:30:30 (**c**) or control vector and CoV-Gluc (**d**) were transfected with CDK2/CyclinA plasmids or CDK1/CyclinB plasmids for 48 hours. CDK1/2 overexpression was determined by Western blot analysis.


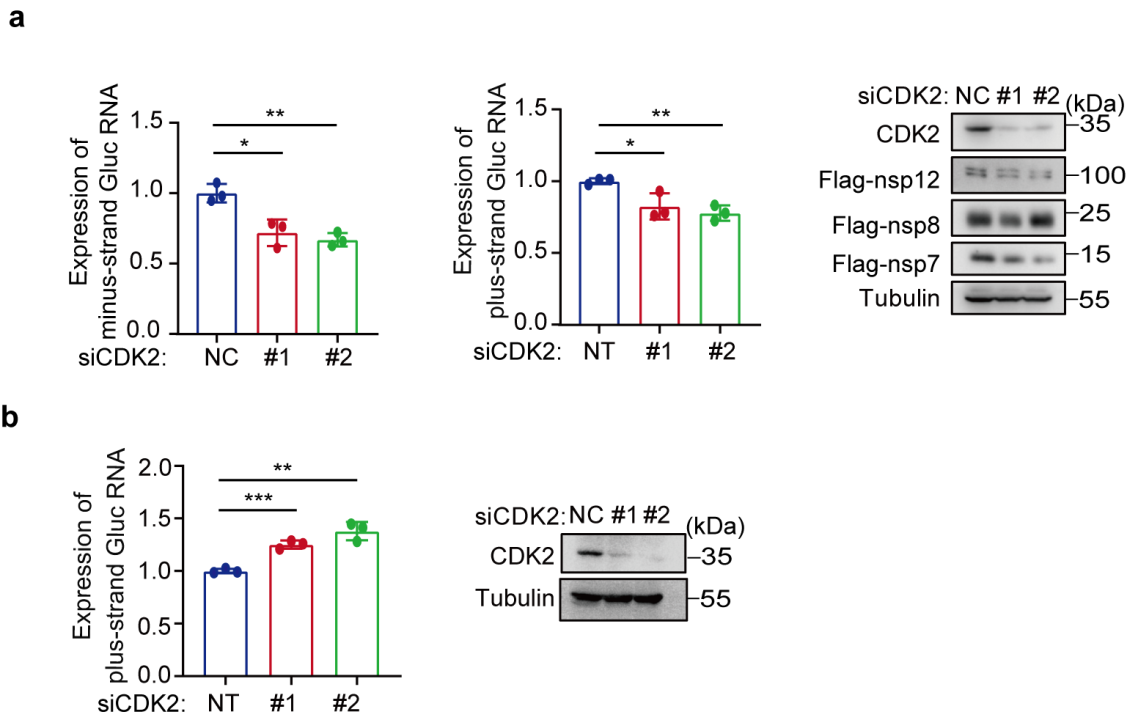


**Figure. S2. CDK2 knockdown specifically impairs the activity of RdRp.** **a, b** Hela cells expressing CoV-Gluc, nsp12, nsp7, nsp8 plasmid DNA at the same ratio as before (**a**) or control vector and CoV-Gluc (**b**) were transfected with two siRNA specific sequence for CDK2. After 48 hours, minus-strand or plus-strand Gluc RNA was detected by qRT-PCR. Cell lysates were analyzed by immunoblot. The experiments were performed in triplicate, and data are shown as mean ± SD, two-tailed unpaired Student’s t-test. *P < 0.05, **P < 0.01, ***P < 0.001.


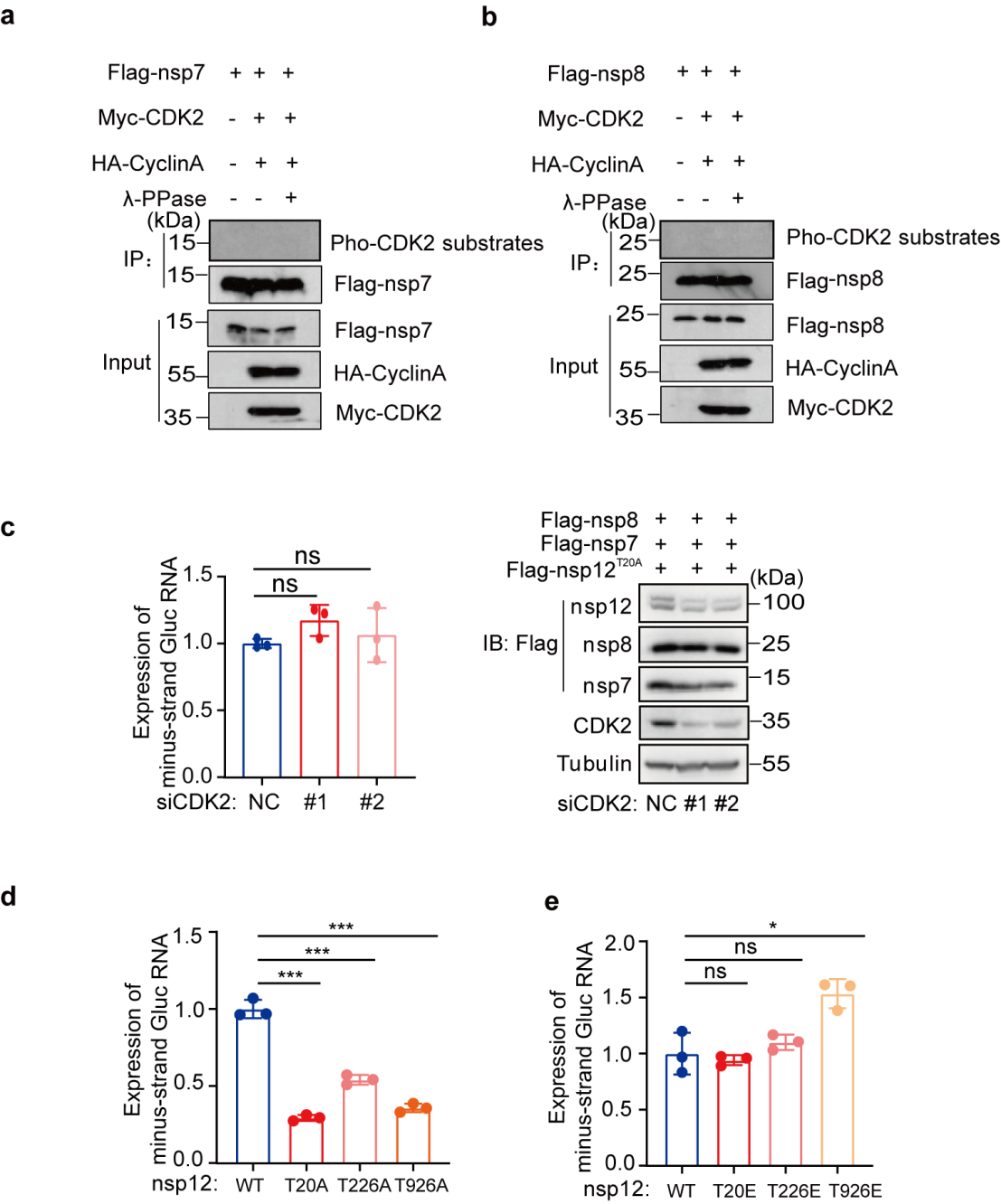


**Figure. S3. CDK2-dependent phosphorylation of RdRp is required for SARS-CoV-2 RNA synthesis.** **a, b** HEK293T cells were transfected with Flag-nsp7 (**a**) or Flag-nsp8 (**b**), Myc-CDK2 and HA-CyclinA for 48 hours, then were treated with/without λ-PPase, followed by immunoblotting with antibodies against Pho-CDK2 substrates and Flag, HA, Myc. **c** HEK293T cells were transfected with individual siRNAs and Flag-nsp7, Flag-nsp8, Flag-nsp12 mutant T20A, and CoV-Gluc plasmids for 48 hours. The minus-strand Gluc-RNA was quantified by qRT-PCR and cell lysates were analyzed by immunoblot. **d, e** Potential CDK2 phosphorylation sites T226 or T926 were predicted by the GPS2.1 software. Gluc-RNA, Flag-nsp7, Flag-nsp8, non-phosphorylated nsp12 mutant (**d**) or phosphor-mimetic nsp12 mutant (**e**) plasmids were transfected in HEK293T cells for 48 hours. The minus-strand Gluc-RNA was quantified by qRT-PCR. Experiment was performed in triplicate, data are shown as mean ± SD, *P < 0.05, ***P < 0.001 and ns, not significant (two-tailed unpaired Student’s t-test).

**
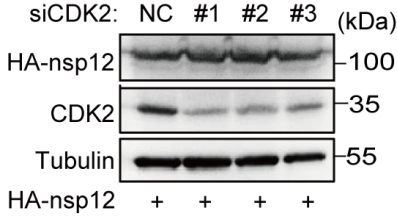
**

**Figure. S4. CDK2 has no significant effect on nsp12 expression.**  Individual siRNAs and HA-nsp12 plasmids were transfected in HEK293T cells respectively and incubated for 48 hours. Cell lysates were analyzed by immunoblotting.


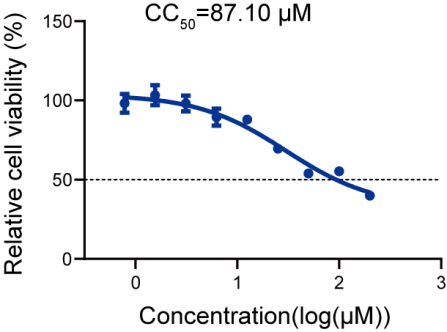


**Figure. S5.** **CC_50_ values of the SNS-032.** CC_50_ values of the SNS-032 were measured in Vero cells with CCK-8 kits. Data showed the average of three independent experiments.

**Table S1**. **Host proteins of molecular weights between 30 kDa and 40 kDa**

| **Accession** | **Gene names** | **MW [kDa]** | **Sequence coverage (%)** |
| --- | --- | --- | --- |
| P13804 | ETFA | 35.06 | 32.73 |
| O14579 | COPE | 34.46 | 26.30 |
| Q9GZS3 | WDR61 | 33.56 | 25.57 |
| Q96FW1 | OTUB1 | 31.26 | 24.35 |
| Q15785 | TOMM34 | 34.54 | 23.30 |
| P19623 | SRM | 33.80 | 23.18 |
| P06493 | CDK1 | 34.07 | 22.56 |
| Q9Y314 | NOSIP | 33.15 | 22.26 |
| Q16762 | TST | 33.41 | 20.54 |
| P11908 | PRPS2 | 34.75 | 20.13 |
| Q9Y316 | MEMO1 | 33.71 | 19.87 |
| P32322 | PYCR1 | 33.34 | 19.75 |
| Q9NTX5 | ECHDC1 | 33.68 | 18.57 |
| Q96C36 | PYCR2 | 33.62 | 17.19 |
| P10768 | ESD | 31.44 | 15.96 |
| P43897 | TSFM | 35.37 | 13.54 |
| P00491 | PNP | 32.10 | 13.49 |
| O75792 | RNASEH2A | 33.37 | 13.04 |
| P24941 | CDK2 | 33.91 | 12.75 |
| Q16836 | HADH | 34.27 | 12.74 |
| P53597 | SUCLG1 | 36.23 | 12.14 |
| Q00535 | CDK5 | 33.28 | 11.99 |
| P63244 | RACK1 | 35.05 | 11.67 |
| P43307 | SSR1 | 32.22 | 11.19 |
| O00165 | HAX1 | 31.60 | 11.11 |
| P36542 | ATP5F1C | 32.98 | 10.74 |
| Q9Y5K5 | UCHL5 | 37.58 | 10.64 |
| P00387 | CYB5R3 | 34.21 | 10.63 |
| Q9Y277 | VDAC3 | 30.64 | 10.60 |
| A6NDG6 | PGP | 33.98 | 10.59 |
| Q9NZD8 | SPG21 | 34.94 | 10.39 |
| Q9NUI1 | DECR2 | 30.76 | 10.27 |
| Q07021 | C1QBP | 31.34 | 9.93 |
| Q9Y6C9 | MTCH2 | 33.31 | 9.90 |
| P05141 | SLC25A5 | 32.83 | 9.73 |
| Q9H2W6 | MRPL46 | 31.69 | 9.68 |
| Q9H936 | SLC25A22 | 34.45 | 8.98 |
| Q53GQ0 | HSD17B12 | 34.30 | 8.97 |
| Q9Y315 | DERA | 35.21 | 8.81 |
| P48507 | GCLM | 30.71 | 8.76 |
| Q9UJZ1 | STOML2 | 38.51 | 8.71 |
| P21912 | SDHB | 31.61 | 8.57 |
| Q9UFF9 | CNOT8 | 33.52 | 8.56 |
| Q8TC12 | RDH11 | 35.36 | 8.49 |
| Q9H3N1 | TMX1 | 31.77 | 7.86 |
| P08758 | ANXA5 | 35.91 | 7.50 |
| P21796 | VDAC1 | 30.75 | 7.42 |
| P30084 | ECHS1 | 31.37 | 7.24 |
| Q9NPF4 | OSGEP | 36.40 | 7.16 |
| P08574 | CYC1 | 35.40 | 6.77 |
| Q96FX7 | TRMT61A | 31.36 | 6.23 |
| Q9BSH4 | TACO1 | 32.46 | 6.06 |
| O00560 | SDCBP | 32.42 | 6.04 |
| Q9Y4U1 | MMACHC | 31.71 | 5.67 |
| O95456 | PSMG1 | 32.83 | 5.56 |
| Q9NPL8 | TIMMDC1 | 32.16 | 5.26 |
| P04818 | TYMS | 35.69 | 5.11 |
| Q9HBH5 | RDH14 | 36.84 | 5.06 |
| Q9H4A6 | GOLPH3 | 33.79 | 5.03 |
| Q9HA47 | UCK1 | 31.41 | 4.69 |
| Q96CX2 | KCTD12 | 35.68 | 4.62 |
| Q9NP79 | VTA1 | 33.86 | 4.56 |
| Q00403 | GTF2B | 34.81 | 4.43 |
| Q14232 | EIF2B1 | 33.69 | 4.26 |
| O95983 | MBD3 | 32.82 | 4.12 |
| Q96NB2 | SFXN2 | 36.21 | 4.04 |
| Q14192 | FHL2 | 32.17 | 3.94 |
| Q53HL2 | CDCA8 | 31.30 | 3.93 |
| Q9NX46 | ADPRS | 38.92 | 3.86 |
| P0C2W1 | FBXO45 | 30.61 | 3.85 |
| Q9UNE7 | STUB1 | 34.83 | 3.63 |
| Q96GK7 | FAHD2A | 34.57 | 3.50 |
| Q15006 | EMC2 | 34.81 | 3.37 |
| P40926 | MDH2 | 35.48 | 3.25 |
| Q9BQP7 | MGME1 | 39.40 | 3.20 |
| Q5HYK3 | COQ5 | 37.12 | 3.06 |
| Q8NBN7 | RDH13 | 35.91 | 3.02 |
| Q9Y399 | MRPS2 | 33.23 | 2.70 |
| Q8TDX7 | NEK7 | 34.53 | 2.65 |
| Q9UHQ9 | CYB5R1 | 34.07 | 2.62 |
| Q3ZCQ8 | TIMM50 | 39.62 | 2.27 |
| O00487 | PSMD14 | 34.55 | 2.26 |
| Q9BTT0 | ANP32E | 30.67 | 2.24 |
| Q16698 | DECR1 | 36.04 | 2.09 |
|  |  |  |  |

Table S2. Primer sequences used for real-time qPCR analysis

| Primer | Sequence |
| --- | --- |
| CoV-Gluc-F | CCGAAAGGTAAGATGGGCGT |
| CoV-Gluc-R | CTTTCTAGCGTTGGCCTCCA |
| CDK1-F | AAACTACAGGTCAAGTGGTAGCC |
| CDK1-R | TCCTGCATAAGCACATCCTGA |
| CDK2-F | CCAGGAGTTACTTCTATGCCTGA |
| CDK2-R | TTCATCCAGGGGAGGTACAAC |
| ETFA-F | CTTCGGAAAGAACCTTTTGCCC |
| ETFA-R | GTTCCACGGACAGAAAACACT |
| OTUB1-F | CTGACGGCAACTGTTTCTATCG |
| OTUB1-R | CAGGTCCATGAACGTGTTGTG |
| CDK5-F | GGAAGGCACCTACGGAACTG |
| CDK5-R | GGCACACCCTCATCATCGT |
| Actin-F | AGAAAATCTGGCACCACACC |
| Actin-R | AGAGGCGTACAGGGATAGCA |

Table S3 Targets for each CDK inhibitor

| **Kinase inhibitor** | **Target** |
| --- | --- |
| SNS-032 (BMS-387032) | CDK2, CDK7, 9 |
| Flavopiridol (Alvocidib) | CDK1, CDK2, CDK4 and CDK6 |
| JNJ-7706621 | CDK1/2 |
| AT7519 | CDK1, 2, 4, 6 and 9 |
| BS-181 HCl | CDK7 |
| BMS-265246 | CDK1/2, CDK4 |
| AZD5438 | CDK1/2/9 |
| Flavopiridol HCl | CDK1, CDK2, CDK4, CDK6, EGFR, PKA |
| R547 | CDK1/2/4 |
| PHA-767491 | Cdc7/CDK9, CDK1/2 and GSK3-β |
| Milciclib (PHA-848125) | CDK2, CDK1, 4, 5, and 7 |
| Dinaciclib (SCH727965) | CDK2, CDK5, CDK1 and CDK9 |
| TG003 | Clk1, Clk2, and Clk4 |
| LDC000067 | CDK9 |
| SU9516 | CDK2, CDK1, and CDK4 |
| Ro-3306 | CDK1 |
| P276-00 | CDK1, CDK4 and CDK9 |
